# Q fever in Spain: Description of a new series, and systematic review

**DOI:** 10.1371/journal.pntd.0006338

**Published:** 2018-03-15

**Authors:** Vanesa Alende-Castro, Cristina Macía-Rodríguez, Ignacio Novo-Veleiro, Xana García-Fernández, Mercedes Treviño-Castellano, Sergio Rodríguez-Fernández, Arturo González-Quintela

**Affiliations:** 1 Department of Internal Medicine, University Teaching Hospital, Santiago de Compostela, A Coruña, Spain; 2 Department of Internal Medicine, Povisa Hospital, Vigo, Pontevedra, Spain; 3 Department of Microbiology, University Teaching Hospital, Santiago de Compostela, A Coruña, Spain; Faculté de Médecine,Aix-Marseille Université, FRANCE

## Abstract

**Background:**

Forms of presentation of Q fever vary widely across Spain, with differences between the north and south. In the absence of reported case series from Galicia (north-west Spain), this study sought to describe a Q-fever case series in this region for the first time, and conduct a systematic review to analyse all available data on the disease in Spain.

**Methods:**

Patients with positive serum antibodies to *Coxiella burnetii* from a single institution over a 5-year period (January 2011-December 2015) were included. Patients with phase II titres above 1/128 (or documented seroconversion) and compatible clinical criterial were considered as having Q fever. Patients with clinical suspicion of chronic Q-fever and IgG antibodies to phase I-antigen of over 1/1024, or persistently high levels six months after treatment were considered to be cases of probable chronic Q-fever. *Systematic review*: We conducted a search of the Pubmed/Medline database using the terms: *Q Fever* OR *Coxiella burnetii* AND *Spain*. Our search yielded a total of 318 studies: 244 were excluded because they failed to match the main criteria, and 41 were discarded due to methodological problems, incomplete information or duplication. Finally, 33 studies were included.

**Results:**

A total of 155 patients, all of them from Galicia, with positive serological determination were located during the study period; 116 (75%) were deemed to be serologically positive patients without Q fever and the remaining 39 (25%) were diagnosed with Q fever. A potential exposure risk was found in 2 patients (5%). The most frequent form of presentation was pneumonia (87%), followed by isolated fever (5%), diarrhoea (5%) and endocarditis (3%). The main symptoms were headache (100%), cough (77%) and fever (69%). A trend to a paucisymptomatic illness was observed in women. Hospital admission was required in 37 cases, and 6 patients died while in hospital. Only 2 patients developed chronic Q-fever. *Systematic review*: Most cases were sporadic, mainly presented during the winter and spring, as pneumonia in 37%, hepatitis in 31% and isolated fever in 29.6% of patients. In the north of Spain, 71% of patients had pneumonia, 13.2% isolated fever and 13% hepatitis. In the central and southern areas, isolated fever was the most frequent form of presentation (40%), followed by hepatitis (38.4%) and pneumonia (17.6%). Only 31.7% of patients reported risk factors, and an urban-environment was the most frequent place of origin. Overall mortality was 0.9%, and the percentage of patients with chronic forms of Q-fever was 2%.

**Conclusions:**

This is the first study to report on a Q-fever case series in Galicia. It shows that in this region, the disease affects the elderly population -even in the absence of risk factors- and is linked to a higher mortality than reported by previous studies. While pneumonia is the most frequent form of presentation in the north of the country, isolated fever and hepatitis tend to be more frequent in the central and southern areas. In Spain, 32% of Q-fever cases do not report contact with traditional risk factors, and around 58% live in urban areas.

## Author summary

We present, for the first time, a series which describes clinical and epidemiological characteristics of patients diagnosed with Q fever in our region (Galicia, North-Western Spain). Our results showed that pneumonia is the main form of presentation of Q fever in our area, with headache and cough as the most frequent symptoms. We also found a very low percentage of patients with potential exposure risks (5%), which reinforces the hypothesis of the existence of alternative mechanisms of transmission to animal reservoir contact. Due to the lack of data available, we believe that the present work could be the starting point to improve the knowledge of this disease in our area. We also developed a systematic review of all series published in Spain, which showed a geographycal pattern linked to form of presentation, with pneumonia as the main form in Northern regions and hepatitis and isolated fever in Central and Southern regions.

## Introduction

Q fever is a zoonosis with a world-wide distribution, caused by *Coxiella burnetii*, which can present as sporadic cases or through outbreaks in a specific region. Although ticks can play a role as vectors [1], the usual sources of human infection are cattle, sheep, goats, and wildlife. Since its description, Q fever has been widely studied by many groups, with the 2008 outbreak in the Netherlands increasing interest in the disease in Europe in recent years [2].

The relevance of Q fever in Spain varies widely depending on the region in question, e.g., whereas large series have been reported in the Basque Country, only a few case reports have come from other regions [3,4]. These differences might be due to climatological, social or environmental factors but for the most part, knowledge of the epidemiology of *Coxiella burnetii* is poor in Spain. To our knowledge there have been no previously reported case series of Q fever in Galicia (north-west Spain), and we only have a limited perspective of the distribution of *Coxiella burnetii* in animal reservoirs [5], along with reports of a few isolated cases [6]. Although the presence of cattle is frequent in all areas of Galicia, the existence of an additional wildlife reservoir is also plausible, because populations of foxes, wolves, wild boars and other species also exist in the region. While existing data tend to suggest a difference in clinical presentation between the north and south of the country [7,8], there are nevertheless insufficient data to describe this in Galicia and elsewhere for the purpose of giving a complete picture of Q fever in Spain.

Accordingly, the main objectives of this study were: on one hand, to describe a Q-fever case series in Galicia for the first time; and on the other, to conduct a systematic review to clarify and analyse all available data on the disease in Spain.

## Results

### Case-series report

A total of 155 patients were located with positive serological determination during the study period; of these, 116 (75%) were deemed to be serologically positive patients without Q fever (unrelated to an acute or chronic *Coxiella burnetii* infection) and the remaining 39 (25%) were diagnosed with Q fever. Regarding the group without Q fever, the indication for serological testing was atypical pneumonia or pneumonia in patients with risk factor for Q fever in 80% of cases, and fever wihtout a clear origin in the remainder. In this second group, main final diagnoses were gastrointenstinal disease, viral infections and neoplasia. All patients included in the group without Q fever had a final diagnosis that excluded Q fever as the cause of their symptoms. Of the Q fever patients, 27 were men (69%), and only 2 (5%) were found to have a potential risk of exposure (contact with cattle), although this data was available only in 50% of patients. A rural environment was the most frequent place of residence (22 patients, 56%). None of the patients had reported a tick bite before the appearance of clinical symptoms. A breakdown of the complete epidemiological profile of the series is shown in Table 1.

**Table 1:** Clinical and epidemiological characteristics of patients and differences between genders.

	Total (n = 39)	Men (n = 27)	Women (n = 12)	*P*
Age	73 (15)	73 (11)	72 (22)	0.839
Animal exposure	2 (5)	0 (0)	2 (17)	-
Rural environment	22 (56)	15 (55.5)	7 (58)	0.872
COPD	9 (23)	9 (33)	0 (0)	0.022
Chronic heart failure	12 (47)	9 (33)	3 (25)	0.450
Chronic liver disease	2 (5)	2 (7)	0 (0)	0.474
Fever	27 (69)	21 (78)	6 (50)	0.089
Headache	39 (100)	27 (100)	12 (100)	-
Dyspnea	23 (59)	20 (74)	3 (25)	0.006
Cough	30 (77)	22 (81.5)	8 (67)	0.268
Chest pain	11 (28)	9 (33)	2 (17)	0.253
Abdominal pain	7 (18)	5 (18.5)	2 (17)	0.635
ESR > 35 mm/h	16 (41)	11 (41)	5 (42)	0.614
CRP > 2 mg/dL	16 (41)	9 (33)	7 (58)	0.133
CRP > 10 mg/dL	9 (23)	7 (26)	2 (17)	0.424
Leukocytes > 10000/μL	24 (62)	17 (63)	7 (58)	0.528
Leukocytes < 5000/μL	4 (10)	4 (15)	0 (0)	0.213
Cytolysis (AST/ALT > 45 IU/L)	10 (26)	8 (30)	2 (17)	0.332
Cholestasis (GGT > 55 IU/L or AP > 150 IU/L)	16 (41)	11 (41)	5 (42)	0.893
Anaemia (Hb < 12 g/dL)	13 (33)	7 (26)	6 (50)	0.135

Data are shown as absolute values (percentage).

COPD: chronic obstructive pulmonary disease. ESR: erythrocyte sedimentation rate. CRP: C reactive protein. AST: aspartate amino-transferase. ALT: alanine amino-transferase. GGT: gamma glutamyl-transferase. AP: alkaline phosphatase. Hb: haemoglobin.

Most cases were diagnosed during 2015 (26 cases, 67%) and 2014 (11 cases, 28%), with all presenting in the period from December to June (winter and spring). In terms of clinical manifestations, the most frequent form of presentation was pneumonia (35 cases, 90%), followed by isolated febrile syndrome (2 cases), diarrhoea (2 cases) and endocarditis (1 case). The main symptom reported by patients was headache (100%), followed by cough (77%) and fever (69%). It is remarkable that a trend to paucisymptomatic illness was observed in women compared with men. All clinical and analytical aspects are shown in Table 1.

Hospital admission in the Internal Medicine or Pneumology departments was required in all but 2 patients, both of whom successfully underwent ambulatory treatment and follow-up. When it came to empirical treatment, the most frequent regimen included a beta-lactam antibiotic (25 patients, 64%), either alone (8 patients) or in combination with a fluoroquinolone (7 patients) or a macrolide (10 patients). After diagnosis of Q fever, 11 patients were switched to doxycycline in monotherapy and another 6 patients to a fluoroquinolone, while the remaining 22 patients completed the initial empirical treatment. There were no differences in patients’ length of hospital stay, duration of symptoms or mortality rates by reference to the treatment received, with the average length of antibiotic therapy being 13 (SD = 6) days.

At the date of diagnosis, 28 patients (72%) had phase II titres of over 1/512. Across follow-up, only 2 patients developed a probable chronic Q fever: one had confirmed endocarditis through transesophageal echocardiogram, and the other had a persistent febrile syndrome which was also judged to be endocarditis, despite it was not possible to perform a complete study due to poor general condition of this patient. Both patients were alive at the end of the study period, and continued undergoing long-term treatment with doxycycline and hydroxychloroquine.

Insofar as mortality was concerned, six patients (15%) died, in all cases due to respiratory complications during hospital stay. No epidemiological, clinical or biochemical variable was found to be linked to mortality in our series.

### Systematic review

This review covered 33 case-series reports (excluding ours) spanning a 38-year period (1977–2014) in Spain. Data on a total of 1960 patients (including our study) were analysed. A breakdown of the main data available from the studies is shown in Table 2.

**Table 2:** Main data obtained from the studies included in the systematic review.

*STUDY* Location	DATE	N	SERIES	CLINICAL PRESENTATION	SYMPTOMS	ENVIRONMENT	RISK FACTORS	BIOCHEMICAL DATA	EVOLUTION
*Hellín et al.[9]* Madrid	Sept 1977—Aug 1980	23	Sporadic	Isolated fever: 14 (61) Pneumonia: 9 (39)	Fever: 23 (100) Headache: 18 (78) Myalgia: 14 (61) Respiratory symptoms: 13 (56)	ND	Animal (livestock) or food exposure: 9 (39)	Transaminase: 17 (74) Leukopenia: 10 (43) Leukocytosis: 2 (9)	Deceased: 0 (0) Chronic: 0 (0)
*Montejo-Baranda et al.[7]* Vizcaya	June 1981- June 1982	11	Sporadic	Pneumonia: 10 (91) Isolated fever: 1 (9)	Fever: 11 (100) Headache: 11 (11) Myalgia: 11 (100)	Urban: 6 (54) Rural: 5 (46)	Non-specific: 4 (36)	Transaminase: 7 (64) Leukopenia: 5 (36)	Deceased: 0 (0) Chronic: 0 (0)
*Aguirre-Errasti et al.[10]* Vizcaya	March—May 1982	42	Epidemic	Pneumonia: 28 (67) Hepatitis: 11 (26)	Fever: 42 (100) Headache: 42 (100) Myalgia: 42 (100)	ND	ND	Transaminase: 16 (38)	Chronic: 0 (0) Deceased: 0 (0)
*Menéndez-Caro et al.[11]* Madrid	1982–1983	26	Sporadic	Isolated fever: 22 (85) Pneumonia: 3 (11) Endocarditis: 1 (4)	Fever: 26 (100) Headache: 18 (69) Asthenia: 16 (61) Chills: 11 (42) Sweating: 8 (31) Cough: 7 (27)	ND	Food exposure: 4 (15) Animal exposure (livestock): 2 (8)	Transaminase: 15 (58) ESR: 8 (31) Leukocytosis: 7 (27) Leukopenia: 7 (27)	Deceased: 1 (4) Chronic: 3 (11)
*Fernández-Roblas et al.[12]* Madrid	1982–1984	37	Sporadic	Isolated fever: 28 (75) Pneumonia: 8 (22) Hepatitis: 1 (3)	Fever: 37 (100) Headache: 27 (73) Chills: 20 (54) Respiratory symptoms: 18 (49) Arthromyalgia: 14 (38)	ND	Non-specific: 12 (32)	Transaminase: 21 (57) Leukopenia: 5 (13.5)	Deceased: 0 (0) Chronic: 5 (13.5)
*Sobradillo et al.[13]* Vizcaya	Jul 1982—Dec 1986	164	Sporadic	Pneumonia: 164 (100)	Fever: 158 (96) Headache: 91 (55) Myalgia: 88 (54) Cough 88 (54) Chest pain: 56 (34)	Urban: 89 (54) Rural: 75 (46)	Animal exposure (ND): 13 (8)	Transaminase: 74 (45) ESR: 71 (43) Leukocytosis: 22 (13)	Deceased: 0 (0) Chronic: 0 (0)
*De Alarcón et al.[8]* Seville	1983–1999	231	Sporadic	Hepatitis: 148 (64) Isolated fever: 75 (32) Pneumonia: 3 (1)	Fever: 231 (100) Chills: 174 (75) Headache: 172 (74.5) Myalgia: 158 (68) Arthralgia: 139 (60) Cough: 41 (18)	Urban: 123 (53) Rural: 108 (47)	Animal exposure (ND): 91 (39) Food exposure: 46 (20) Rural stay: 19 (15)	Transaminase: 139 (60) Thrombocytosis: 43 (19) Thrombopenia: 41 (18) Leukopenia: 23 (10) Leukocytosis: 16 (7)	Chronic: 0 (0) Deceased: 0 (0)
*Martínez-Luengas et al.[14]* Seville	Sept 1983—Oct 1984	34	Sporadic	Pneumonia: 11 (32) Isolated fever: 22 (65)	Fever: 34 (100) Headache: 32 (94) Arthromyalgia: 31 (91)	ND	Non-specific: 20 (59)	Transaminase: 17 (50) ESR: 14 (41) Leukocytosis: 5 (15)	Chronic: 1 (3) Deceased: 0 (0)
*Merino et al.[15]* Soria	Jan 1984—Dec 1996	13	Sporadic	Pneumonia: 10 (77) Isolated fever: 1 (8) Hepatitis: 1 (8)	Fever: 13 (100) Cough: 4 (31) Headache: 3 (23)	ND	Animal exposure (ND): 2 (16)	ND	Chronic: 1 (8) Deceased: 0 (0)
*Domingo et al.[16]* Barcelona	Jan 1985—Dec 1997	63	Sporadic	Hepatitis: 30 (48) Pneumonia: 26 (41) Isolated fever: 7 (11)	Fever: 63 (100)	Urban: 63 (100)	Animal exposure (pets): 30 (48)	ND	Deceased: 0 (0) Chronic: 0 (0)
*Antón-Aranda et al.[17]* Guipúzcoa	1985–1989	60	Sporadic	Pneumonia: 44 (73) Hepatitis: 17 (28) Isolated fever: 7 (12)	Fever: 60 (100) Arthromyalgia: 38 (63) Cough: 33 (55) Headache: 32 (53)	ND	Animal (livestock) or food exposure: 10 (17)	Transaminase: 28 (47) ESR: 17 (28) LDH: 11 (18) Leukocytosis: 11 (18) Thrombopenia: 11 (18)	Deceased: 0 (0) Chronic: 1 (2)
*Millán-Mon et al.[18]* La Palma Canary Islands	1986–1988	35	Sporadic	Isolated fever: 26 (74) Hepatitis: 7 (20) Pneumonia: 2 (6)	Fever: 35 (100) Headache: 16 (46) Cough: 9 (26) Arthromyalgia: 13 (37)	ND	Animal exposure (goats): 18 (51)	Transaminase: 31 (88) ESR: 27 (77) Leukocytosis: 9 (26)	ND
*Romero-Jiménez et al.[19]* Huelva	1987–1999	109	Sporadic	Hepatitis: 60 (55) Isolated fever: 47 (43)	Fever: 106 (97) Headache: 83 (76) Arthromyalgia: 48 (44)	Urban: 10 (9) Rural: 99 (91)	Animal exposure (ND): 57 (52)	Leukopenia: 36 (33) Thrombopenia: 28 (26)	ND
*Abad et al.[20]* Vizcaya	Jan 1988—Jan 1998	73	Sporadic	Pneumonia: 55 (75) Isolated fever: 18 (25)	Fever: 73 (100) Headache: 33 (45) Cough: 30 (41)	ND	Non-specific: 11 (15)	Transaminase: 33 (45) Leukocytosis: 5 (7)	Deceased: 0 (0) Chronic: 0 (0)
*Rotaeche del Campo et al.[21]* Guipúzcoa	Feb 1989	5	Epidemic	Pneumonia: 3 (60) Isolated fever: 2 (40)	Fever: 5 (100) Myalgia: 5 (100) Headache: 4	Rural: 5 (100)	Animal exposure (ND): 5 (100)	ESR: 3 (60) Transaminase: 2 (40)	ND
*Bella et al.[22]* Barcelona	1989	17	Sporadic	Pneumonia: 4 (23) Hepatitis: 12 (71) Isolated fever: 1 (6)	Fever: 17 (100)	Urban: 17 (100)	ND	Transaminase: 14 (82)	ND
*Sampere et al.[23]* Barcelona	1989–1999	66	Sporadic	Pneumonia: 37 (56) Hepatitis: 22 (33) Isolated fever: 5 (7.5) Pleuropericarditis: 1 (1.5) Bronchitis: 1 (1.5)	Fever: 52 (79) Headache: 28 (42) Arthromyalgia: 27 (41)	Urban: 66 (100)	Animal exposure (ND): 24 (36) Rural stay: 8 (12)	Transaminase: 40 (61) Cholestasis: 29 (44)	Deceased: 0 (0) Chronic: ND
*Martínez-Eizaguirre et al.[24]* Guipúzcoa	April—June 1990	30	Epidemic	Isolated fever: 30 (100)	Fever: 30 (100) Sweating: 29 (97) Headache: 26 (87) Chills: 25 (83) Myalgia: 22 (73)	Rural: 30 (100)	ND	Transaminase: 26 (86) ESR: 5 (17) Leukocytosis: 4 (13) Leukopenia: 4 (13)	Deceased: 0 (0) Chronic: ND
*Pascual-Velasco et al.[25]* Lanzarote Canary Islands	1991–1992	94	Sporadic	Pneumonia: 52 (55) Isolated fever: 42 (45)	ND	ND	Animal exposure (goats): 25 (27)	ND	ND
*Muñoz-Sanz et al.[26]* Badajoz	June 1992—May 2005	124	Sporadic	Isolated fever: 64 (52) Hepatitis: 53 (43) Pneumonia: 14 (11) Endocarditis: 7 (6)	Fever: 120 (97) Headache: 40 (32)	Urban: 48 (39) Rural: 76 (61)	Animal exposure (sheep, goats and cows): 58 (47)	ND	Deceased: 0 (0) Chronic: 7 (6)
*Espejo et al.[27]* Canary Islands—La Rioja—Catalonia	1995–2009 (Canary Islands 2005–2009)	183	Sporadic	Hepatitis: 90 (49) Isolated fever: 58 (32) Pneumonia: 35 (19)	Headache: 107 (58.5) Arthromyalgia: 69 (38) Nausea/vomiting: 32 (17.5)	Urban: 120 (65.5) Rural: 63 (34.5)	ND	ND	Deceased: 2 (1) Chronic: ND
*Nuño-Mateo et al.[28]* Asturias	Feb 1996—May 2001	12	Sporadic	Pneumonia: 10 (83) Isolated fever: 2 (17)	Headache: 12 (100) Fever: 12 (100) Cough: 8 (66)	Rural: 3 (25) Urban: 9 (75)	Animal exposure (sheep and cattle): 5 (42)	Transaminase: 11 (90) ESR: 11 (90)	Deceased: 0 (0) Chronic: 0 (0)
*Lepe et al.[29]* Huelva	1996–1997	21	Sporadic	Isolated fever: 19 (90) Pneumonia: 2 (10)	Fever: 21 (100) Headache: 21 (100) Myalgia: 21 (100)	ND	Animal exposure (ND): 9 (43)	Transaminase: 21 (100)	Deceased: 0 (0) Chronic: 0 (0)
*Bartolomé et al.[30]* Albacete	April 1997—Sept 2002	35	Sporadic	Hepatitis: 17 (48) Pneumonia: 9 (26) Isolated fever: 8 (23) Myocarditis: 1 (3)	Fever: 34 (95) Headache: 16 (46) Arthromyalgia: 13 (37) Cough: 12 (34)	Urban: 25 (71.5) Rural: 10 (28.5)	Animal exposure (livestock): 9 (26) Rural stay: 5 (14)	Transaminase: 26 (74) ESR: 14 (40)	Deceased: 1 (3) Chronic: ND
*Nebreda et al.[31]* Soria	May 1998	14	Epidemic	Pneumonia: 9 (64) Isolated fever: 5 (36)	Fever: 13 (93) Headache: 10 (71) Sweating: 8 (57) Myalgia: 5 (36)	Rural: 14 (100)	Animal exposure (ND): 3 (21) Food exposure: 1 (7) Rural stay: 2 (14)	Leukopenia: 2 (14)	Chronic: 0 (0) Deceased: 0 (0)
*Bolaños et al.[32]* Gran Canaria Canary Islands	1998–2000	59	Sporadic	Hepatitis: 52 (88) Pneumonia: 7 (12) Isolated fever: 3 (5)	Fever: 59 (100)	Urban: 32 (54) Rural: 27 (46)	Animal exposure (ND): 15 (25)	Transaminase: 52 (88) APTT: 26 (44)	Deceased: 1 (2) Chronic: 0 (0)
*Martín-Aspas et al.[33]* Cádiz	2000–2010	80	Sporadic	Hepatitis: 50 (63) Isolated fever: 16 (20)	Fever: 78 (98)	Urban: 69 (86) Rural: 11 (14)	ND	Transaminase: 43 (54) Thrombocytopenia: 39 (49)	Deceased: ND Chronic: 16 (20)*
*Ramos et al.[4]* Alicante	Jan 2001—Sept 2004	30	Sporadic	Hepatitis: 21 (70) Isolated fever: 5 (17) Pneumonia: 3 (10) Meningo-encephalitis: 1 (3)	Fever: 30 (100) Headache: 29 (97) Myalgia: 25 (83)	Urban: 20 (67) Rural: 10 (33)	Animal exposure (ND): 16 (53)	CRP: 29 (97) Transaminase: 20 (67) ESR: 19 (63) LDH: 17 (51.5) APTT: 12 (36)	Deceased: 0 (0) Chronic: ND
*Ruiz-Seco et al.[34]* Madrid	2001–2008	54	Sporadic	Pneumonia: 29 (54) Hepatitis: 13 (24) Isolated fever: 13 (24)	Fever: 37 (68) Cough: 25 (47) Breathless: 13 (25)	Urban: 28 (52) Rural: 26 (48)	Animal exposure (ND): 32 (59)	ESR: 37 (72) Leukopenia: (60) Thrombocytosis: (60)	Chronic: 3 (8) Deceased: 3 (8)
*García-Clemente et al.[35]* Asturias	Jan/Feb 2003	60	Epidemic	Pneumonia: 60 (100)	Fever: 60 (100) Headache: 48 (80) Myalgia: 46 (77) Cough: 42 (70)	Rural: 29 (48) Urban: 31 (52)	Animal exposure (livestock): 4 (7)	Transaminase: 21 (35) APTT: 14 (24)	Deceased: 0 (0) Chronic: 0 (0)
*Raya-Cruz et al.[36]* Mallorca Balearic Islands	March 2003—Feb 2011	87	Sporadic	Pneumonia: 39 (45) Isolated fever: 21 (24) Hepatitis: 19 (22) Pericarditis: 3 (3.5) Meningitis: 1 (1) Meningo-encephalitis: 1 (1)	Fever: 62 (71) Cough: 32 (37) Headache: 23 (26) Breathless: 15 (17)	Urban: 35 (40) Rural: 39 (45) Undefined: 13 (15)	Animal exposure (cattle, sheep and goats): 8 (9) Urban animal exposure (pets): 31 (36) Food exposure: 1 (1)	Leukocytosis: 27 (31) Transaminase: 29 (33)	Chronic: 1 (1) Deceased: 3 (3.4)
*De los Ríos-Martín et al.[37]* Madrid	Feb—March 2004	16	Epidemic	Isolated fever: 14 (87.5) Pneumonia: 2 (12.5)	Fever: 16 (100) Anorexia: 13 (81) Asthenia: 11 (69) Cough: 9 (56)	ND	Animal exposure (ND): 16 (100)	Transaminase: 2 (12.5) Thrombopenia: 2 (12.5)	ND
*Alonso et al.[38]* Vizcaya	Feb—April 2014	50	Epidemic	Isolated fever: 14 (28) Pneumonia: 13 (26)	Fever: 27 (54) Headache: 22 (44) Myalgia: 11 (22)	Urban: 44 (88) Rural: 6 (12)	Animal exposure (livestock): 3 (6)	ND	Chronic: 0 (0) Deceased: 0 (0)

Data are shown as absolute values (percentage).

* All patients diagnosed with chronic Q fever on the basis of biochemical criteria alone.

CRP: C reactive protein; ESR: erythrocyte sedimentation rate; LDH: lactate dehydrogenase; APTT: activated partial thromboplastin time; ND: no data.

It was noteworthy that, when the overall data (including those of our series) were analysed, most of the cases were seen to be sporadic, presenting mainly during winter and spring, as pneumonia in 37%, hepatitis in 31% and isolated fever in 29.6% of cases. Furthermore, on pooling the case series from areas located in the northern third of the country and comparing these to the remaining series from central and southern Spain, a significant difference was seen in the form of presentation of Q fever. In the northern areas, 71% of patients had pneumonia, followed by isolated fever (13.2%) and hepatitis (13%), while in the central and southern areas isolated fever was the most frequent form of presentation (40%), followed by hepatitis (38.4%) and pneumonia (17.6%).

With regard to clinical manifestations, fever was the most frequent (94.6%), followed by headache (50.2%), and myalgia or arthromyalgia (33.5%). Mention should be made of the absence of fever in 108 cases (5.4%), despite diagnosis of Q fever. A risk factor, mainly animal exposure (in most of cases livestock, but also pets), was identified in only 31.7% of patients, and urban environment was the most frequent place of origin (833/1469, 58%), though these data were missing in 12 studies.

Overall mortality was 0.9% but the absence of mortality data in 8 studies should be noted. The total percentage of patients with chronic forms of Q fever was 2%, yet it has to be said that no data were reported in 11 studies, and in one study 16 cases were reported as chronic on the basis of serological criteria alone [33].

## Discussion

To our knowledge, this is the first Q fever case-series study to have been conducted in Galicia (north-west Spain), an area not previously considered endemic. In this respect, our data show a significant increase in diagnosis of Q fever in recent years, with a peak in 2015. Even so, we observed no evidence of geographical clustering or contact between cases that would justify our series being classified as an outbreak. A high percentage (75%) of patients with positive serological determination were deemed to be serologically positive patients without Q fever, in most cases (80%) due to low titres in the context of a community-acquired pneumonia caused by other bacteria. In respect of these patients, there are two important aspects that could represent potential limitations: on the one hand, there was no serological follow-up in any case to exclude late seroconversion; and on the other, our data did not contain the necessary information on *Coxiella burnetii* seroprevalence to evaluate these results. Further analysis is thus called for. The main limitation of our series report is the restrospective design, which limitates information about exposures and specific clinical data.

Analysis of the clinical presentation of the cases included shows that the high percentage of pneumonia observed by us is consistent with the findings reported by other studies conducted in the north of Spain [8,17,39] but not with those reported by studies conducted in the south [8,14,19]. The reasons for this difference between northern and southern areas of the country are not completely understood. Recent studies suggest that different *Coxiella burnetii* genotypes coexist and infect different wildlife species in Spain [40]. Hence, one could hypothesize that different bacterial genotypes might lead to different clinical presentations of Q fever. Further studies are needed to clarify this potential relationship.

Our study observed a difference between men and women in the prevalence of respiratory symptoms and fever, a finding consistent with recent reports [41]. While this difference could be due to a higher prevalence of chronic obstructive pulmonary disease among men, or, by the same token, to a different inflammatory response in women. In this connection, a different prevalence of adverse effects to *Coxiella burnetii* vaccine in men and women has recently been reported [42].

The presence of some cases without fever which were nonetheless diagnosed as Q fever is clearly striking. In our series, this group accounted for 31% of patients, and while this percentage is similar to that reported by previous studies in Madrid [34], the Balearic Islands [36] and the Basque Country [38], it is not the usual pattern [7–10,13,14,16,17,20,22,24,26,32]. As the above three were the most recently published case-series studies prior to ours, diagnosis of cases without fever could reflect the greater reliability of new diagnostic methods, or even a higher level of suspicion among physicians vis-à-vis patients who present with symptoms and signs compatible with Q fever, due to better knowledge of this disease and its epidemiology. The absence of fever in a third of the patients in our series could be due to a higher prevalence of pneumonia caused by *Coxiella burnetii*, which can be suspected and diagnosed without fever [43]. In our series, all patients without fever (12) had pneumonia, which was also the main clinical presentation in the series from Madrid and the Balearic Islands [34,36]. These findings are in line with those reported by studies undertaken elsewhere [44,45] and may reflect the usefulness of performing Q fever serological tests on all patients with community-acquired pneumonia [44].

With regard to risk factors, these were found in only 2 patients (5%). Moreover, nearly half of the cases lived in urban areas, theoretically less exposed to contact with *Coxiella burnetii*. This lack of association with traditional risk factors for Q fever was confirmed by our systematic review, which showed a higher percentage of patients from urban areas, and the presence of risk factors in only a third of all patients [7,8,13]. This same finding has been observed in studies conducted at different periods in different geographical areas [7–9,13,19,26,36]. The first explanation to be considered is the high percentage of missing data on risk factors in most of the studies analysed, something that could clearly lead to such factors being underestimated [10,18,24,27]. Other possible explanations are linked to different mechanisms of transmission, such as aerosols in drinking water [46], consumption of unpasteurised milk products [47], or contact with ticks and other vectors [48], which might not have been investigated in all patients. Recent studies also suggest the possibility of long-distance dispersion of *Coxiella burnetii* by wind [49], transmission through waste manipulation [38], or even human transmission [50] as novel mechanisms that might not have been considered in most series. At all events, in future detailed investigation of potential risk factors should be made standard practice in all patients diagnosed with Q fever, to clarify all possible transmission mechanisms.

In terms of biochemical markers, as in other studies, the most frequent alteration in our series was elevation of the leukocyte count and liver enzyme levels, even in the absence of cases presented as hepatitis [8,20,24].

Only two cases were diagnosed as probable chronic Q fever, one which initially presented as endocarditis and another which presented as a persistent febrile syndrome. Serological follow-up was performed in only a third of patients, something that might represent a potential study limitation. Previous studies reported similar percentages, probably due to the same limitation [13,17,32]. Martín-Aspas *et al* found a higher proportion of patients who fulfilled the serological criteria for chronic Q fever 3 months after diagnosis, but none of them met the clinical criteria for complete diagnosis of chronic Q fever [33]. This study underscores the vital importance of long-term follow-up in patients diagnosed with Q fever, regardless of the duration or type of treatment received, and puts into question the usefulness of serological monitoring, in which analysis has previously yielded contradictory results [51,52]. A regular, full clinical evaluation thus remains the main tool for diagnosis of chronic forms of Q fever, and the follow-up period should be no less than 6 months [53]. Other diagnostic tests, like Positron Emission Tomography (PET, not available in our series), have showed an increasing utility in the diagnosis of Q fever, particularly in persistent forms [54].

Another remarkable finding was the mortality observed in our series (15%), in that it is the highest Q fever-related mortality rate reported in Spain, according to our systematic review. Possible explanations for this difference might lie in a higher prevalence of comorbidity and an older cohort. In this regard, most studies reported cases in younger adult populations, mean age 30–40 years [8–10,13,14,16–20,22,30,37,38], whereas the age range in our study extended up to 73 years. Furthermore, the prevalence of previous chronic diseases was also higher in our series, in which 20 patients (51%) had at least one chronic disease. In contrast, previous studies reported a comorbidity prevalence of around 20% [32,35,36], which may have contributed to a less severe disease profile and a lower mortality rate. Lastly, our series included patients who required hospitalisation in 95% of cases, which implies a greater severity and a higher risk of complications. Other authors included a variable percentage of outpatients, ranging from 20–40% [20,32] to 80% [10,38], which probably means the presence of less severe forms of Q fever and, by extension, lower mortality. Another plausible explanation could be the existence of a more virulent clone or genotype of *Coxiella burnetii* in our area, which would require further studies to be demonstrated [41].

### Conclusions

To the best of our knowledge, this is the first Q fever case-series study to have been conducted in Galicia (north-west Spain), an area that had not been considered endemic until now. Our study shows that the presence of Q fever in this area is relevant, affects the elderly population even in the absence of risk factors, and is linked to a higher mortality than previous studies have indicated. Our systematic review confirms the different presentation of Q fever in northern areas of Spain, where pneumonia is the most frequent form, compared to central and southern areas, where isolated fever and hepatitis are more frequent. This systematic review also shows that nearly 32% of Q fever cases do not report contact with traditional risk factors, and that around 58% of them reside in urban areas. Better knowledge of seroprevalence in different areas and a higher level of clinical suspicion are therefore needed, in order to improve diagnosis and prevention of this disease in Spain.

## Materials and methods

### Case-series report

A search of the Microbiology Unit database at the Santiago de Compostela University Teaching Hospital (A Coruña, Galicia, Spain), covering a 4-year period from January 2011 to December 2015, located a total of 155 patients. We reviewed the clinical histories of all patients with positive serological determination. In terms of area of residence, “urban” was defined as any area having a population of over 50000, “suburban” as any area having a population of 5000 to 50000, and “rural” as any area having a population of less than 5000.

IgG antibodies to phase I and phase II *Coxiella burnetii* antigens were determined by indirect immunofluorescence [55,56], according to the manufacturer's instructions (Vircell, Spain). Assayed dilutions began from 1/64 onwards. Seroconversion was considered in all patients with phase-II titres of over 1/128 who had previously been seronegative, or in those who showed a fourfold or greater rise in IgG antibody titre to *Coxiella burnetii* phase II antigen (corresponding to 2 dilutions) [57,58]. All titres above 1/128 were deemed to be positive, and we investigated the clinical history of all patients with these serological levels.

Clinical, epidemiological, microbiological, radiological and biochemical variables were collected by reviewing patients’ electronic medical records to check for diagnosis of acute Q fever. Inclusion criteria were defined as: positive serological determination; or alternatively, documented seroconversion and clinical diagnosis of acute Q fever, based on compatible signs and symptoms and exclusion of other causes. Patients with a positive serological test but a confirmed aetiology for clinical manifestations other than Q fever were excluded and classified as serologically positive patients without Q fever. Patients with clinical suspicion of chronic Q fever (cardiac valve disease, prosthetic or bone infection, immunocompromised hosts, or persistent fever of unknown origin) and IgG antibodies to phase-I antigen of over 1/1024, or persistently high levels (over 1/800) six months after treatment were considered to be cases of probable chronic Q fever [59]. Patients with criteria for probable chronic Q fever and vegetations in trasesophageal echocardiogram and/or compatible findings after pathological examination of cardiac valves after surgery were considered as Q fever endocarditis [41].

As regards clinical presentation, patients were classified as having: pneumonia, on presentation of radiological consolidation and fever; hepatitis, on presentation of transaminase elevation more than twofold the upper limit of normal; and fever of unknown origin, if they displayed no other clinical, radiological or biochemical alterations.

### Ethics statement

The study protocol was reviewed and approved by the Galician Clinical Research Ethics Committee. All data analyzed were anonymized.

### Systematic review

We conducted a systematic review in order to identify observational studies published before December 2015, describing Q fever case series in Spain. To this end, we searched the Pubmed/Medline database using the following terms: *Q Fever* OR *Coxiella burnetii* AND *Spain*. In addition, we retrieved additional series by surveying references cited in other reports and using the MEDLINE option, "Related articles".

These search methods yielded a total of 318 studies; after a critical perusal of titles and abstracts, 244 were excluded for failing to fulfil the main eligibility criteria (series reports with a complete clinical description). A critical, full-text review of the remaining 74 papers led to a further 41 being discarded because of methodological problems, incomplete clinical information or series duplication. Finally, 33 studies were included in the systematic review.

### Statistical methods

A descriptive analysis was performed, by calculating qualitative-variable rates along with their mean and standard deviation. We used the Chi-square or Fisher's exact tests, as appropriate (expected frequency value <5), to compare qualitative variables, and the Student's t test for quantitative variables. A *P*-value <0.05 was regarded as significant. All analyses were performed using the SPSS v. 22.0 computer software package (SPSS Inc., Chicago, IL, USA).

## Supporting information

S1 Checklist STROBE checklist. (DOC)

S2 Checklist PRISMA checklist. (DOC)

S1 Flowchart PRISMA flowchart. (DOC)

S1 Database Cases database. (XLS)
