# Supplementary material for: Q fever in Spain: Description of a new series, and systematic review
Source: PLoS Negl Trop Dis. 2018 Mar 15;12(3):e0006338. doi: 10.1371/journal.pntd.0006338 (PMC5871012; doi:10.1371/journal.pntd.0006338)
Supplement: S1 Flowchart — (DOC) [file pntd.0006338.s003.doc]

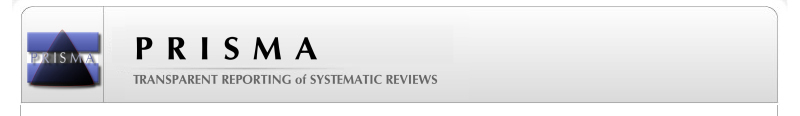
**PRISMA 2009 Flow Diagram**

**Screening**

**Included**

**Eligibility**

**Identification**

Records identified through database searching
(n = 219 )

Additional records identified through other sources
(n = 99 )

Records after duplicates removed
(n = 298 )

Records screened
(n = 298 )

Records excluded due to methodological reasons
(n = 21 )

Full-text articles assessed for eligibility
(n = 277 )

Full-text articles excluded after applied inclusion criteria (n = 244 )

Studies included in qualitative synthesis
(n = 33 )

Studies included in quantitative synthesis (meta-analysis)
(n = N/A )
